# Three New Mitochondrial Genomes of Semisulcospiridae J. P. E. Morrison, 1952 (Caenogastropoda: Cerithioidea) from China and Insights into Their Phylogenetic Position

**DOI:** 10.3390/genes16121488

**Published:** 2025-12-12

**Authors:** Yibin Xu, Yuanzheng Meng, Sheng Zeng, Deyuan Yang, Shen Zhong, Zeyang Lin, Xiaohong Chen, Zhao Zhang, Hangjun Wang, Huidong Zheng

**Affiliations:** 1 Key Laboratory of Cultivation and High-Value Utilization of Marine Organisms in Fujian Province, Fisheries Research Institute of Fujian, Xiamen 361013, China; yibinxu@163.com (Y.X.);; 2 College of the Environment and Ecology, Xiamen University, Xiamen 361005, China; markmeng200001@gmail.com (Y.M.); deyuanyang92@163.com (D.Y.);; 3 School of Marine Biology, Xiamen Ocean Vocational College, Xiamen 361012, China; 4 Wenzhou Marine Center, Ministry of Natural Resources of the People’s Republic of China, Beijing 100804, China; 5 Marine Ecosystem Observation and Research Station on the Yangtze River Estuary, Wenzhou 325011, China

**Keywords:** freshwater gastropods, systematics, phylogenomics, comparative mitogenomics

## Abstract

**Background:** Semisulcospiridae is a family of freshwater gastropods with over 100 species, primarily distributed in East Asia and North America. They play crucial ecological roles and are of medical importance as intermediate hosts for parasites. However, their phylogenetic relationship remains unclear. Most previous studies, which focused on fewer molecular markers (e.g., *COI*, *16S*, *28S*), have shown limitations in resolving relationships with low resolution. Mitochondrial genomes, with their richer phylogenetic information, offer a promising tool to infer the evolutionary relationships within this family. **Methods:** This study sequenced, assembled, and annotated the complete mitochondrial genomes of three Semisulcospiridae species from China: *Koreoleptoxis friniana*, *Hua textrix*, and *Hua yangi*. Phylogenetic analyses were conducted using Maximum Likelihood (ML) and Bayesian Inference (BI) methods on five distinct datasets derived from the mitochondrial genomes, including nucleotide sequences of protein-coding genes (with and without third codon positions), amino acid sequences, and combinations with two ribosomal RNA genes. **Results:** The complete (or near-complete) mitochondrial genomes of *K. friniana*, *H. textrix*, and *H. yangi* were 15,474 bp, 15,660 bp, and 15,744 bp in length, respectively, showing typical gene content and an A+T bias. The gene order was highly conserved. Phylogenetic analyses consistently recovered the family Semisulcospiridae as monophyletic and revealed three well-supported, distinct clades corresponding to the genera *Semisulcospira*, *Koreoleptoxis*, and *Hua*. While the overall tree topologies were robust for Semisulcospiridae, some incongruences were observed in the placements of other cerithioidean families depending on the dataset used. Evolutionary rate analysis (Ka/Ks) indicated strong purifying selection across all protein-coding genes, with *COX1* being the most conserved. **Conclusions:** This study provided three new mitochondrial genomes for Semisulcospiridae: *K. friniana*, *H. textrix*, and *H. yangi.* Phylogenetic analysis based on mitochondrial genome datasets offers new evidence that supports the monophyly of the three Asian genera of Semisulcospiridae. Future research should include broader taxonomic sampling, particularly of the North American genus *Juga* and the atypical Japanese *Semisulcospira* lineages, to achieve a comprehensive phylogenetic framework.

## 1. Introduction

Semisulcospiridae Morrison, 1952 is a family of freshwater gastropods, comprising over 100 species from four genera [1]. The members of this family are mainly distributed in East Asia and North America, with the majority (over 60 species of three genera) recorded in China [2,3]. They inhabit various freshwater bodies, including rivers, lakes, streams, and springs [2,3,4,5,6,7]. Semisulcospirids have attracted considerable research interest due to their important ecological roles and their role as intermediate hosts of human pathogens [3,5,6,7,8]. For instance, many semisulcospirids prefer clean, well-oxygenated waters, making them useful indicators of environmental quality [3,6,7]. Moreover, several species serve as intermediate hosts for medically significant trematodes [9,10,11]. Given their ecological and medical significance, a comprehensive understanding of their phylogenetic relationships is crucial [5,6].

Currently, there are four genera recognized under Semisulcospiridae. *Hua* S.-F. Chen, 1943, *Koreoleptoxis* J. B. Burch & Y. Jung, 1988, and *Semisulcospira* O. Boettger, 1886 are distributed in Asia and *Juga* H. Adams & A. Adams, 1854 in America [5,12]. *Semisulcospira* and *Koreoleptoxis* are mainly distributed in the northern and central regions of China, and *Hua* is distributed in the southwest of China [2,5]. The genus *Semisulcospira* is distinguished from other genera by its reproductive mode, because it is the only viviparous species in Semisulcospiridae. *Koreoleptoxis* and *Hua* can be distinguished by their female reproductive organs: female *Hua* species have an egg-laying groove or an ovipositor under the left tentacle, while *Koreoleptoxis* has both structures [2]. There are at least 13 species of *Koreoleptoxis* recorded in China, and almost all species of *Hua* are recorded in and endemic to China, except *H. jacqueti*, which is also recorded in Vietnam [2,13]

The phylogenetic relationships within Semisulcospiridae remain unresolved. Strong and Köhler [13] were the first to revise the taxonomy of this family using both morphological characteristics and molecular evidence. Köhler [6,7] subsequently provided comprehensive systematic treatments of *Semisulcospira* species from Japan and Korea, and Du et al. (2019a, 2019b, 2023) further investigated the phylogeny of Chinese semisulcospirids [2,4,5]. However, these studies relied mainly on a limited number of mitochondrial and nuclear markers, such as partial *COI*, 16S rRNA, and 28S rRNA genes. Three genera, *Juga*, *Hua*, and *Koreoleptoxis*, are generally monophyletic according to the phylogenetic trees based on those molecular markers [4,5,12,14], while the phylogenetic position of *Semisulcospira* is still in mystery. However, the problems mentioned above suggest that traditionally used molecular markers showed limitations in solving the phylogeny of Semisulcospiridae. Therefore, more methods have been applied to solve this question. Sawada and Fuke [15] imported genome-wide SNPs and have been successful in resolving the systematic relationships of the Japanese semisulcospirids [15,16,17,18,19]. Xu et al. [14,18] applied mitochondrial genomes in the phylogenetics of Semisulcospiridae with the description of a new species.

Animal mitochondrial DNA (mtDNA) is a cornerstone molecular marker in evolutionary biology. This compact, circular genome, typically 14–20 kb in length, encodes 37 genes, including 13 protein-coding genes (PCGs), two ribosomal RNAs (rRNAs), and 22 transfer RNAs (tRNAs) [20]. Its maternal inheritance, lack of recombination, and relatively high evolutionary rate have made it indispensable for studies in species identification, population genetics, and phylogenetic reconstruction across diverse taxonomic scales [21]. Therefore, we believe that more comprehensive sampling may help to clarify the phylogenetic relationships of Semisulcospiridae.

So far, the mitochondrial genome data of Semisulcospiridae reported in NCBI only include eight different species from three genera (four of *Semisulcospira*, three of *Koreoleptoxis,* and only one of *Hua*). In this study, we reported another three species from two genera of Semisulcospiridae from China: *K. friniana* (Heude, 1888 [22]), *H. textrix* (Heude, 1889 [23]), and *H. yangi* (Du et al., 2023 [2]). The former is common in the Southeast of China, sometimes consumed as food by local people, and the latter two species are endemic to China. We hope that studying the mitochondrial genome data of these species will improve our understanding of the phylogeny of Semisulcospiridae and thereby assist with research in conservation biology, ecology, and public health.

## 2. Materials and Methods

### 2.1. Specimen Collection, Identification, and Sequencing

The sequenced specimen was collected from freshwater bodies in China. The specimen was fixed in ethanol (≥95%), preserved in a −20 °C refrigerator, and finally deposited at the College of the Environment and Ecology, Xiamen University. DNA extraction and library preparation protocols followed the procedures described in Yang et al. [24]. The morphological and molecular information (*COI* and *16S* rRNA, see Supplementary File S2, Table S1) was integrated for species identification. The information of the specimens used in this study is shown in Table 1.

Identification followed by Heude [22,23], Du et al. [5], Du and Yang [2], and Zeng et al. [25]. Some of the specimens used in this study are the same as those in Zeng et al. [25].

**Figure 1:**
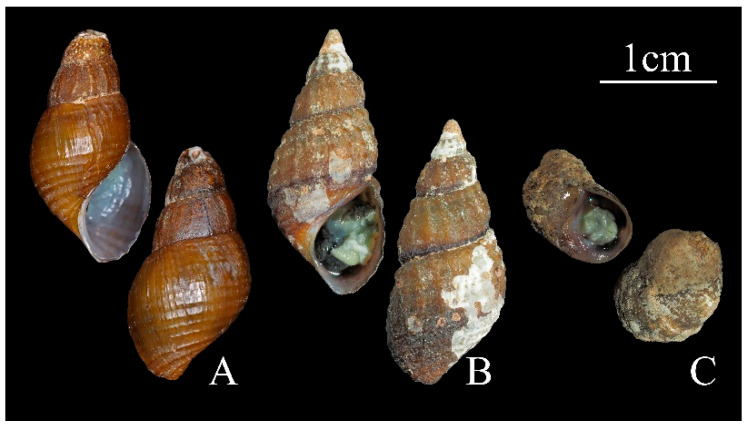
Specimens sequenced in this study. (**A**) *Koreoleptoxis friniana* (Heude, 1888 [22]), RTM11; (**B**) *Hua textrix* (Heude, 1889 [23]), SEM-A1; (**C**) *Hua yangi* Du et al., 2023 [2], SEM-B1.

***K. friniana* (Heude, 1888** [22]**), RTM11 (Figure 1A):** Shell medium-sized, solid, conical, with seven whorls. Surface brown, with 13–16 spiral lines and 13–15 weak axial ribs crossing each other, forming checkerboard patterns. Apex seriously eroded. The blast results show high identity values (over 99%) with *COI* sequences of *K. friniana* (MK969058, MK969049, etc.) [4].

***H. textrix* (Heude, 1888 [22]), SEM-A1 (Figure 1B):** Shell medium-sized, solid, conical, with seven whorls. Surface brown, with a dark brown band on the bottom of each whorl, with eight spiral raised lines and 13–16 axial ribs crossing each other, Apex pointed. Aperture ovate. The blast results show high identity values (over 99%) with two *COI* sequences and two 16S of *H. textrix* (MK251701, MK969005; MK251613, MK251612) [4,5].

***H. yangi* Du et al., 2023 [2], SEM-B1 (Figure 1C):** Shell small, solid, ovate, with four whorls. Surface brown, smooth, often covered by algae and sediment. Body whorl inflated. Apex blunt. Aperture round. The blast results show high identity values (over 99%) with *COI* sequences of *H. yangi* (MK969082, MK969071, etc.) [2,5].

### 2.2. Assembly and Annotation of Mitochondrial Genome

Raw paired-end sequencing reads were quality-filtered using Fastp v.0.23.4 [26] to remove adapter sequences and trim low-quality regions [27]. Mitochondrial genome assembly was performed using GetOrganelle v.1.7.6.1 [28] with K-mer sizes of 17, 21, 33, 39, 45, 55, 65, 75, 85, 95, 105, 115, and 127; all other parameters were set to default values.

The assembled mitochondrial genomes were annotated using MitoFinder v.1.4.1 [29] and MitoZ v.3.6 [30]. The resulting annotation files (GenBank format) were reorganized using PhyloSuite v.1.2.3 [31] with *cox1* (*COI*, *COX1*) designated as the starting gene. Annotations were subsequently manually refined in Geneious Prime v.2022.2.2 [32], adhering to the annotation principles outlined by Yang et al. [24]. The annotated mitochondrial genome sequences are provided in Supplementary File S1.

### 2.3. Phylogenetic Analysis

Phylogenetic analyses incorporated the dataset from Xu et al. (2024) [27], supplemented with newly sequenced mitochondrial genomes of *K. friniana* (RTM11), *H. textrix* (SEM-A1), and *H. yangi* (SEM-B1) (Supplementary File S2, Table S1). Protein-coding genes (PCGs) and two ribosomal RNA genes (2R) were extracted and aligned using MAFFT under normal mode settings. Ambiguously aligned regions were removed using TrimAl v.1.2 [33] with the ‘automated1’ setting (Supplementary File S2, Table S2).

Phylogenetic reconstructions were performed on five distinct datasets: (i) 13PCGs123, encompassing all three codon positions of the 13 PCGs; (ii) 13PCGs123+2R, combining the 13PCGs dataset with two rRNA genes; (iii) 13PCGs12, excluding third codon positions of the 13 PCGs; (iv) 13PCGs12+2R, combining the 13PCGs12 dataset with two rRNA genes; and (v) 13PCGsAA, comprising amino acid sequences translated from the 13 PCGs.

Optimal substitution models were determined using ModelFinder v.2.2.0 (Kalyaanamoorthy et al., 2017 [34]) under partitioned schemes: the Bayesian Information Criterion (BIC) for maximum likelihood (ML) analysis and the corrected Akaike Information Criterion (AICc) for Bayesian inference (BI) analysis (Supplementary File S2, Table S3).

Maximum likelihood phylogenetic reconstruction was conducted using IQ-TREE v.2.2.2 [34,35] with the optimal partition scheme and edge-linked partition model. Node support values were assessed using 100,000 ultrafast bootstrap replicates. Bayesian inference was performed in MrBayes v.3.2.7a [36] with two independent runs of 1,000,000 generations each. Convergence was evaluated by monitoring the average standard deviation of split frequencies (ASDSF), with additional generations performed if ASDSF exceeded 0.005.

Topological incongruences among the inferred phylogenies were evaluated using TreeSpace [37] implemented in R v.4.3.1 [38], which utilizes Metric Multidimensional Scaling (MDS) to visualize tree relationships and hierarchical clustering methods (including single linkage, complete linkage, UPGMA, and Ward’s method) to identify distinct phylogenetic clusters formally. Final ML and BI trees were visualized using iTOL v.6 [39].

### 2.4. Sequence Analyses

Circular representations of mitochondrial genomes were generated using Proksee v.1.0.0a6 (https://proksee.ca/) [40] Strand asymmetries were quantified using the formulas of Perna and Kocher [41]: AT-skew = (A − T)/(A + T) and GC-skew = (G − C)/(G + C). Codon usage patterns and relative synonymous codon usage (RSCU) values for the 13 protein-coding genes were calculated using PhyloSuite and visualized with the ‘ggplot2’ package [42] in R v.4.1.3 [38]. Non-synonymous (Ka) to synonymous (Ks) substitution rate ratios were estimated using DnaSP v.6.0 [43] for all Semisulcospiridae sequences listed in Supplementary File S2, Table S1.

## 3. Results

### 3.1. Mtgenome Organization

**Figure 2:**
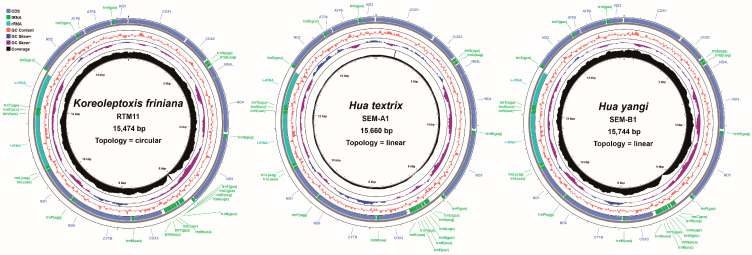
Gene maps of the *K. friniana*, *H. textrix*, and *H. yangi* mtgenomes. The innermost and middle circles depict the distribution of the sequencing depth, GC content, and GC-skew, respectively. The outermost circle represents the arrangement of genes: outer genes from the forward strand, and inner genes from the reverse strand.

We successfully assembled complete mitochondrial genomes from three species: *K. friniana* (15,474 bp), *H. textrix* (15,660 bp), and *H. yangi* (15,744 bp) (Figure 2, Table 2). The genome sequences are deposited in Supplementary File S1. The overall A+T content was 66.0% (*K. friniana*), 65.3% (*H. textrix*), and 64.8% (*H. yangi*) (Table 3). These values are consistent with the AT bias observed across Semisulcospiridae mitochondrial genomes, which range from 64.85% to 77.32% (Supplementary File S2, Table S1).

When calculated from the coding strand of individual genes, the A+T content of the 13 protein-coding genes was 65.4% (*K. friniana*), 64.5% (*H. textrix*), and 64.1% (*H. yangi*) (Table 3). Among the protein-coding genes (PCGs), ATP8 exhibited the highest A+T content, ranging from 71.0% to 72.8% across the three species (*K. friniana*: 72.8%, *H. textrix*: 72.2%, *H. yangi*: 71.0%), while COX3 displayed the lowest, varying from 59.1% to 60.9% (*K. friniana*: 60.9%, *H. textrix*: 59.1%, *H. yangi*: 59.8%) (Table 2).

Both AT-skew and GC-skew values were negative across all three species. AT-skew values followed the pattern: −0.043 (*K. friniana*) > −0.047 (*H. yangi*) > −0.065 (*H. textrix*). GC-skew values demonstrated the order: −0.026 (*H. textrix*) > −0.065 (*H. yangi*) > −0.069 (*K. friniana*).

### 3.2. Genes and Codon Usage

Among the 13 protein-coding genes, all utilized the standard initiation codon ATG, except ND4 in *H. yangi*, which employed GTG as the start codon (Table 2). All 13 genes terminated with canonical stop codons (TAG or TAA). Transfer RNA gene lengths varied from 62 to 72 bp. The 16S rRNA gene length was 1342 bp (*K. friniana*), 1339 bp (*H. textrix*), and 1333 bp (*H. yangi*), while the 12S rRNA gene was 890 bp (*K. friniana*), 892 bp (*H. textrix*), and 896 bp (*H. yangi*) (Table 2). The non-coding region (NCR), located between *trn*F and *trn*C, was 159 bp (*K. friniana*), 312 bp (*H. textrix*), and 391 bp (*H. yangi*).

**Figure 3:**
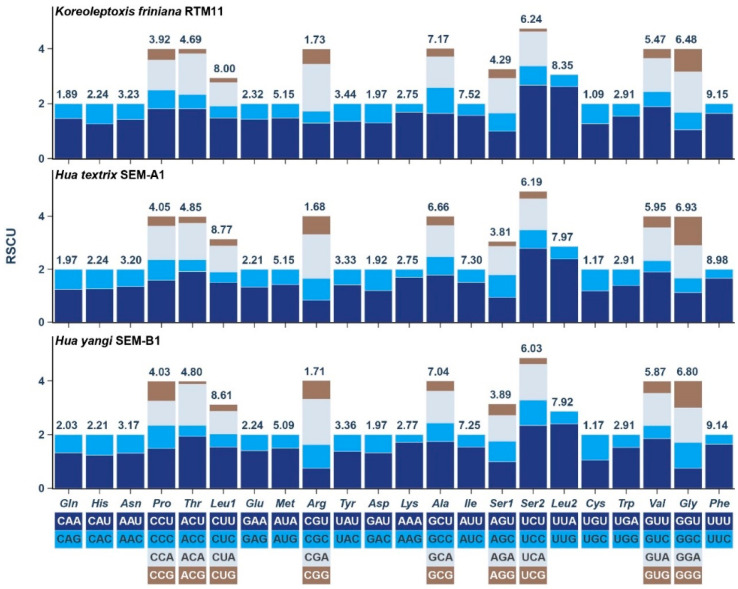
Relative synonymous codon usage (RSCU) of the three mtgenomes. The codon families are provided under the *x*-axis. The frequency of amino acid usage is listed above the column.

Relative synonymous codon usage (RSCU) analysis revealed that UUA (Leucine) exhibited the highest RSCU values across all three species: 2.62 (*K. friniana*), 2.38 (*H. textrix*), and 2.40 (*H. yangi*). The lowest RSCU values were observed for UCG (Serine; 0.12) in *K. friniana*, AGG (Serine; 0.19) in *H. textrix*, and ACG (Threonine; 0.11) in *H. yangi* (Figure 3, Supplementary File S2, Table S4).

### 3.3. Phylogenetic Analysis

**Figure 4:**
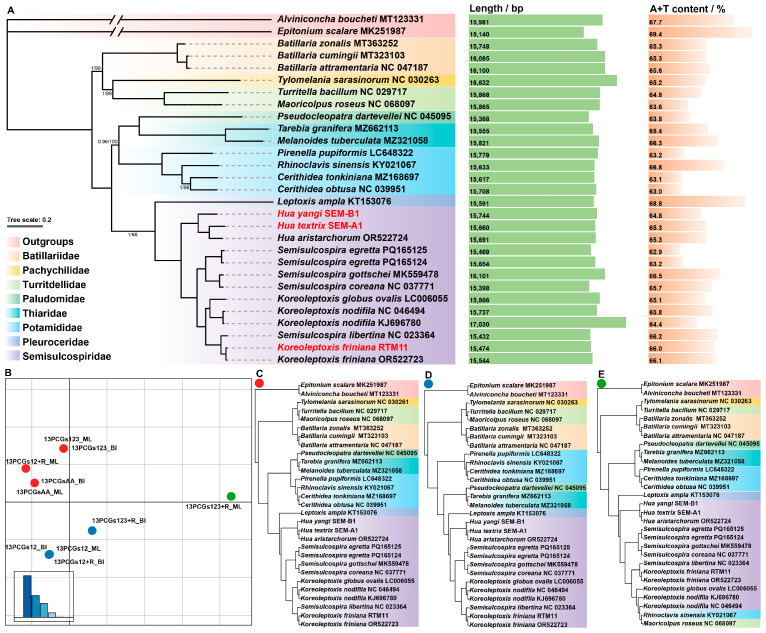
An analysis of phylogenies from five mtgenome datasets. (**A**) Maximum likelihood (ML) and Bayesian inference (BI) tree of Cerithioidea based on the dataset 13PCGs123. The GenBank accession numbers or specimen voucher numbers used are listed after the species names. The scale bar (0.2) corresponds to the estimated number of substitutions per site. Numbers at nodes are statistical support values for “BI posterior probabilities/ML bootstrap support”. “Unlabeled” denotes 100% bootstrap support. Color-coded clades are different families within Cerithioidea. The length and A+T content are shown to the right (full genome). (**B**) Two-dimensional metric multidimensional scaling (MDS) plot of ten trees, colored by different clusters. (**C**–**E**) For each cluster identified in (**B**), a representative tree was selected. Note: Sequences analyzed in this study are highlighted in red in (**A**).

A total of ten maximum likelihood (ML) and Bayesian inference (BI) trees were inferred from five mitogenome datasets of Cerithioidea. These ten trees were grouped into three clusters, representing three distinct topological types (see Figure 4B–E). The most common topology is shown in Figure 4A,C. The primary differences between Cluster 1 (Figure 4C) and Cluster 2 (Figure 4D) lie in the clade formed by *Pseudocleopatra dartevellei* NC_045095, *Tarebia granifera* MZ662113, and *Melanoides tuberculata* MZ321058 (Figure 4). In Cluster 3 (Figure 4E), *Rhinoclavis sinensis* KY021067 and *Maoricolpus roseus* NC_068097 are embedded within Semisulcospiridae sequences, resulting in a substantially different topology from the first two clusters.

The topologies of the phylogenetic trees inferred from 13 PCGs contain 27 Cerithioidea mitogenome sequences, of which 13 belong to Semisulcospiridae. The results demonstrate that Semisulcospiridae is most closely related to Pleuroceridae. The Semisulcospiridae sequences comprise five *Semisulcospira* species, five *Koreoleptoxis* species (including one newly reported in this study), and three *Hua* species (including two newly reported in this study). Four *Semisulcospira* sequences clustered in a clade, and another clade, sister to the former, contains five *Koreoleptoxis* sequences along with a misidentified ‘*Semisulcospira libertina*’ sequence (see Xu et al. [27]). Three *Hua* species formed a clade that is sister to the former two groups.

Regarding the three sequences provided in this study, *H. textrix* SEM-A1 is most closely related to *Hua aristarchorum* OR522724, while *H. yangi* SEM-B1 is sister to the clade formed by the former two sequences. *K. friniana* RTM11 is most closely related to the misidentified ‘*Semisulcospira libertina*’ sequence.

### 3.4. Non-Synonymous/Synonymous Analysis

**Figure 5:**
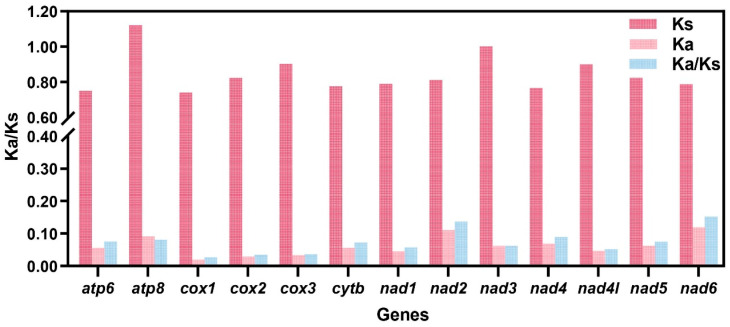
Ka/Ks rates of 13 PCGs based on Semisulcospiridae species. The red, pink, and bule columns represent the values of Ks, Ka, and Ka/Ks, respectively.

Non-synonymous to synonymous substitution ratio (Ka/Ks) analysis demonstrated that *COX1* (Ka/Ks = 0.026), *COX2* (0.035), *COX3* (0.037), and *ND4L* (0.051) evolved at relatively slow rates, indicating strong purifying selection. In contrast, *ND6* (0.152), *ND2* (0.137), *ND4* (0.089), and *ATP8* (0.081) exhibited comparatively elevated evolutionary rates (Figure 5).

## 4. Discussion

### 4.1. Phylogenetic Relationships Within Semisulcospiridae

The phylogeny of Semisulcospiridae has been difficult to resolve, with studies based on a few molecular markers like *COI* and *16S* often yielding unstable topologies and low support values at critical nodes [6,7,14]. Notably, some of these studies suggested a polyphyletic *Semisulcospira* genus, primarily due to the unstable positions of lineages from Japan characterized by unusual branch lengths [6,7]. To assess whether mitochondrial genomes could provide a more stable phylogenetic framework, we conducted phylogenomic analyses. For the species in our sampling, our results robustly support that there are three monophyletic groups corresponding to the three genera, *Hua*, *Semisulcospira*, and *Koreoleptoxis*. This finding is consistent with our previous studies [14,27] (considering the sequence *Semisulcospira libertina* NC_023364 is a misidentification of *Koreoleptoxis* sp., see Xu et al. [27]). A direct comparison with previous studies that reported a polyphyletic *Semisulcospira* genus is constrained by differences in taxonomic coverage, as our current dataset does not include the specific Japanese lineages.

### 4.2. Utility of Mitochondrial Genomes for Phylogenetic Inference

The reliability of the phylogenetic relationships within Semisulcospiridae inferred in this study is underscored by their stability across diverse analytical conditions. Our study generated ten phylogenetic trees from five distinct mitochondrial genome datasets, which were categorized into three topological clusters (Figure 4). Cluster 1 (Figure 4C), containing the most datasets, was selected as the main graph (Figure 4A). Importantly, despite these differences in the placement of other families (e.g., Thiaridae and Paludomidae), the relationships within Semisulcospiridae remained consistent across all clusters. This consistency indicates that the phylogenetic signal from mitochondrial genomic data is robust for this family, irrespective of the specific dataset or analytical approach employed here. Furthermore, we observed that the mitochondrial gene arrangement is highly conserved across the studied Semisulcospiridae species. This conservation suggests that for resolving phylogenetic relationships within this family, the nucleotide sequence information itself plays a far more critical role than gene order comparisons.

### 4.3. Future Directions

While mitochondrial genomes have demonstrated considerable utility in stabilizing the phylogeny for a significant portion of Semisulcospiridae, this study does not single-handedly resolve all historical controversies. The aforementioned Japanese *Semisulcospira* lineages and the North American genus *Juga* represent critical missing taxa in our analysis. Therefore, future research must prioritize the sequencing of mitochondrial genomes from these key groups. Integrating these data will be essential to rigorously test whether the three-clade framework we propose holds universally. Moreover, while nuclear genomic data would be invaluable for an independent phylogenetic assessment, such resources remain scarce for Semisulcospiridae. In this context, and given that our mitogenomic approach provides a more stabilized framework with higher support values compared with single-gene markers, the growing database of mitochondrial genomes presents a practical and promising path forward for future systematic studies aimed at achieving a comprehensive understanding of the family’s evolutionary history.

## Figures and Tables

**Table 1:** Collect information on three semisulcospirid species.

Species	Specimen Catalog	Collection Station	Type Locality	Collection Date
*Koreoleptoxis friniana* (Heude, 1888 [22])	RTM11	Majin River, Kaihua County, Quzhou City, Zhejiang Province, China	Kiangxi = Wuyuan County, Jiangxi Province	September 2022
*Hua textrix* (Heude, 1889 [23])	SEM-A1	Dianchi Lake, Kunming, Yunnan Province, China	Ta-Kouan ho = Daguan River, Kunming, Yunnan Province	September 2023
*Hua yangi* Du et al., 2023 [2]	SEM-B1	Luoping County, Qujing, Yunnan Province, China	Datian River, Zhenfeng County, Guizhou Province	September 2023

**Table 2:** Features of three species’ mtgenomes (RTM11/SEM-A1/SEM-B1).

Genes	Position		Size	Intergenic Nucleotides	Codon		Strand
	From	To			Start	Stop	
*COX1*	1/1/1	1533/1533/1533	1533/1533/1533	25/32/32	ATG/ATG/ATG	TAA/TAA/TAA	H/H/H
*COX2*	1559/1566/1566	2245/2255/2255	687/690/690	24/19/20	ATG/ATG/ATG	TAA/TAA/TAA	H/H/H
*trnS(uga)*	2270/2275/2276	2336/2341/2342	67/67/67	9/10/10			H/H/H
*trnQ(uug)*	2346/2352/2353	2414/2419/2423	69/68/71	31/24/27			H/H/H
*ND4L*	2446/2444/2451	2736/2734/2741	291/291/291	−7/−7/−7	ATG/ATG/ATG	TAA/TAA/TAA	H/H/H
*ND4*	2730/2728/2735	4097/4095/4102	1368/1368/1368	32/33/34	ATG/ATG/GTG	TAA/TAA/TAA	H/H/H
*trnH(gug)*	4130/4129/4137	4195/4194/4201	66/66/65	0/0/0			H/H/H
*ND5*	4196/4195/4202	5914/5913/5920	1719/1719/1719	1/2/2	ATG/ATG/ATG	TAA/TAA/TAA	H/H/H
*trnF(gaa)*	5916/5916/5923	5980/5983/5992	65/68/70	159/312/391			H/H/H
*trnC(gca)*	6140/6296/6384	6205/6357/6446	66/62/63	2/1/3			L/L/L
*trnR(ucg)*	6208/6359/6450	6273/6426/6517	66/68/68	20/21/23			L/L/L
*trnA(ugc)*	6294/6448/6541	6360/6515/6608	67/68/68	16/20/21			L/L/L
*trnN(guu)*	6377/6536/6630	6447/6607/6698	71/72/69	6/7/8			L/L/L
*trnW(uca)*	6454/6615/6707	6520/6682/6773	67/68/67	6/9/9			L/L/L
*trnE(uuc)*	6527/6692/6783	6592/6756/6847	66/65/65	2/2/2			L/L/L
*trnY(gua)*	6595/6759/6850	6661/6825/6917	67/67/68	4/0/0			L/L/L
*trnK(uuu)*	6666/6826/6918	6735/6894/6987	70/69/70	67/68/69			L/L/L
*COX3*	6803/6963/7057	7582/7742/7836	780/780/780	3/3/3	ATG/ATG/ATG	TAA/TAA/TAA	L/L/L
*trnM(cau)*	7586/7746/7840	7652/7815/7908	67/70/69	8/8/9			L/L/L
*CYTB*	7661/7824/7918	8800/8963/9057	1140/1140/1140	−47/−47/−47	ATG/ATG/ATG	TAG/TAA/TAA	L/L/L
*ND6*	8754/8917/9011	9305/9468/9562	552/552/552	2/2/2	ATG/ATG/GTG	TAA/TAA/TAA	L/L/L
*trnP(ugg)*	9308/9471/9565	9372/9536/9630	65/66/66	0/3/3			L/L/L
*ND1*	9373/9540/9634	10,311/10,478/10,572	939/939/939	0/0/0	ATG/ATG/ATG	TAG/TAA/TAA	L/L/L
*trnL(uaa)*	10,312/10,479/10,573	10,379/10,545/10,639	68/67/67	13/16/14			L/L/L
*trnL(uag)*	10,393/10,562/10,654	10,461/10,631/10,723	69/70/70	0/0/0			L/L/L
*l-rRNA*	10,462/10,632/10,724	11,803/11,970/12,056	1342/1339/1333	0/0/0			L/L/L
*trnV(uac)*	11,804/11,971/12,057	11,870/12,039/12,125	67/69/69	2/6/5			L/L/L
*trnG(ucc)*	11,873/12,046/12,131	11,939/12,114/12,197	67/69/67	0/1/1			L/L/L
*trnT(ugu)*	11,940/12,116/12,199	12,007/12,182/12,266	68/67/68	−2/−2/−2			L/L/L
*s-rRNA*	12,006/12,181/12,265	12,895/13,072/13,160	890/892/896	62/61/63			L/L/L
*trnS(gcu)*	12,958/13,134/13,224	13,027/13,201/13,291	70/68/68	0/0/0			H/H/H
*ND2*	13,028/13,202/13,292	14,095/14,269/14,359	1068/1068/1068	−1/0/−2	ATG/ATG/ATG	TAA/TAA/TAG	H/H/H
*trnD(guc)*	14,095/14,270/14,358	14,163/14,338/14,426	69/69/69	4/3/3			H/H/H
*ATP8*	14,168/14,342/14,430	14,329/14,503/14,591	162/162/162	13/9/8	ATG/ATG/ATG	TAA/TAG/TAG	H/H/H
*ATP6*	14,343/14,513/14,600	15,038/15,208/15,295	696/696/696	7/2/2	ATG/ATG/ATG	TAG/TAA/TAA	H/H/H
*trnI(gau)*	15,046/15,211/15,298	15,114/15,282/15,368	69/72/71	1/1/1			H/H/H
*ND3*	15,116/15,284/15,370	15,469/15,637/15,723	354/354/354	4/22/20	ATG/ATG/ATG	TAA/TAG/TAA	H/H/H

**Table 3:** Composition and skewness of three species’ mtgenomes (RTM11/SEM-A1/SEM-B1).

Regions	Size (bp)	T (U)	C	A	G	AT (%)	GC (%)	GT (%)	AT Skewness	GC Skewness
Full genome	15,474/15,660/15,744	34.4/34.8/33.9	18.1/17.8/18.7	31.6/30.5/30.9	15.8/16.9/16.4	66.0/65.3/64.8	33.9/34.7/35.1	50.2/51.7/50.3	−0.043/−0.065/−0.047	−0.069/−0.026/−0.065
PCGs	11,289/11,292/11,292	38.9/38.7/38.2	18.3/18.4/18.9	26.5/25.8/25.9	16.3/17.0/17.0	65.4/64.5/64.1	34.6/35.4/35.9	55.2/55.7/55.2	−0.190/−0.200/−0.193	−0.057/−0.039/−0.052
tRNAs	1486/1495/1495	32.6/31.8/31.4	15.3/16.3/16.7	32.6/31.9/32.8	19.6/20.1/19.0	65.2/63.7/64.2	34.9/36.4/35.7	52.2/51.9/50.4	0.000/0.002/0.022	0.124/0.105/0.064
rRNAs	2232/2231/2229	32.4/32.1/31.9	14.4/15.1/14.8	34.6/34.8/34.2	18.6/18.0/19.2	67.0/66.9/66.1	33.0/33.1/34.0	51.0/50.1/51.1	0.033/0.040/0.035	0.128/0.087/0.128
1st codon position	3763/3764/3764	31.4/30.8/30.8	17.7/18.7/18.5	27.5/27.0/26.9	23.3/23.6/23.8	58.9/57.8/57.7	41.0/42.3/42.3	54.7/54.4/54.6	−0.066/−0.066/−0.067	0.137/0.117/0.125
2nd codon position	3763/3764/3764	43.5/44.0/43.7	22.0/21.7/21.8	18.1/17.9/18.0	16.4/16.4/16.4	61.6/61.9/61.7	38.4/38.1/38.2	59.9/60.4/60.1	−0.412/−0.421/−0.416	−0.143/−0.137/−0.141
3rd codon position	3763/3764/3764	41.9/41.4/40.2	15.1/15.0/16.3	33.9/32.6/32.7	9.1/11.1/10.8	75.8/74.0/72.9	24.2/26.1/27.1	51.0/52.5/51.0	−0.105/−0.119/−0.102	−0.246/−0.149/−0.203
*ATP6*	696/696/696	41.4/43.1/42.5	19.5/18.4/18.2	23.3/22.7/22.8	15.8/15.8/16.4	64.7/65.8/65.3	35.3/34.2/34.6	57.2/58.9/58.9	−0.280/−0.310/−0.301	−0.106/−0.076/−0.054
*ATP8*	162/162/162	40.1/40.1/38.9	16.0/14.2/16.0	32.7/32.1/32.1	11.1/13.6/13.0	72.8/72.2/71.0	27.1/27.8/29.0	51.2/53.7/51.9	−0.102/−0.111/−0.096	−0.182/−0.022/−0.106
*COX1*	1533/1533/1533	36.3/36.9/36.4	19.3/19.3/20.0	26.0/24.1/25.0	18.4/19.7/18.7	62.3/61.0/61.4	37.7/39.0/38.7	54.7/56.6/55.1	−0.166/−0.209/−0.186	−0.024/0.010/−0.034
*COX2*	687/690/690	35.7/36.5/36.7	19.1/18.4/18.6	27.4/27.7/27.1	17.9/17.4/17.7	63.1/64.2/63.8	37.0/35.8/36.3	53.6/53.9/54.4	−0.132/−0.138/−0.150	−0.031/−0.028/−0.024
*COX3*	780/780/780	37.4/35.8/36.2	18.6/19.9/19.6	23.5/23.3/23.6	20.5/21.0/20.6	60.9/59.1/59.8	39.1/40.9/40.2	57.9/56.8/56.8	−0.229/−0.210/−0.210	0.049/0.028/0.025
*CYTB*	1140/1140/1140	39.1/38.2/37.9	19.8/20.3/20.5	24.9/25.1/23.9	16.1/16.4/17.6	64.0/63.3/61.8	35.9/36.7/38.1	55.2/54.6/55.5	−0.222/−0.208/−0.226	−0.102/−0.105/−0.076
*ND1*	939/939/939	42.0/38.8/41.0	15.3/18.0/16.0	25.2/26.2/25.6	17.5/17.0/17.5	67.2/65.0/66.6	32.8/35.0/33.5	59.5/55.8/58.5	−0.249/−0.193/−0.232	0.065/−0.027/0.045
*ND2*	1068/1068/1068	41.9/42.5/41.1	14.8/14.6/15.9	27.4/24.8/25.8	15.9/18.1/17.1	69.3/67.3/66.9	30.7/32.7/33.0	57.8/60.6/58.2	−0.208/−0.263/−0.228	0.037/0.106/0.037
*ND3*	354/354/354	42.1/40.1/38.4	15.0/15.5/18.9	26.8/24.6/25.4	16.1/19.8/17.2	68.9/64.7/63.8	31.1/35.3/36.1	58.2/59.9/55.6	−0.221/−0.240/−0.204	0.036/0.120/−0.047
*ND4*	1368/1368/1368	38.7/38.8/37.7	19.1/19.4/20.1	28.4/27.7/27.6	13.8/14.1/14.5	67.1/66.5/65.3	32.9/33.5/34.6	52.5/52.9/52.2	−0.155/−0.167/−0.154	−0.160/−0.157/−0.160
*ND4L*	291/291/291	37.5/38.8/36.8	17.9/16.2/17.5	26.8/26.5/26.1	17.9/18.6/19.6	64.3/65.3/62.9	35.8/34.8/37.1	55.4/57.4/56.4	−0.166/−0.189/−0.169	0.000/0.069/0.056
*ND5*	1719/1719/1719	38.0/37.9/36.5	19.9/19.4/20.7	28.2/27.8/28.2	13.9/14.8/14.6	66.2/65.7/64.7	33.8/34.2/35.3	51.9/52.7/51.1	−0.148/−0.154/−0.129	−0.177/−0.134/−0.173
*ND6*	552/552/552	39.5/39.7/40.0	16.7/17.2/16.1	27.0/26.1/25.4	16.8/17.0/18.5	66.5/65.8/65.4	33.5/34.2/34.6	56.3/56.7/58.5	−0.188/−0.207/−0.224	0.005/−0.005/0.068
*l-rRNA*	1342/1339/1333	32.5/32.0/32.0	14.2/15.1/14.6	36.0/35.8/34.7	17.3/17.0/18.8	68.5/67.8/66.7	31.5/32.1/33.4	49.8/49.0/50.8	0.051/0.056/0.041	0.097/0.060/0.124
*s-rRNA*	890/892/896	32.2/32.3/31.7	14.6/15.1/15.1	32.6/33.2/33.5	20.6/19.4/19.8	64.8/65.5/65.2	35.2/34.5/34.9	52.8/51.7/51.5	0.005/0.014/0.027	0.169/0.123/0.135

## Data Availability

The original contributions presented in this study are included in the article/Supplementary Materials. Further inquiries can be directed to the corresponding authors.

## Supplementary Materials

The following supporting information can be downloaded at: https://www.mdpi.com/article/10.3390/genes16121488/s1, Supplementary File S1: Three mtigenomes’ information: RTM-11, SEM-A1, and SEM-B1; Supplementary File S2: Supplemental tables; Table S1: The blast results of mtgenome, *COX1* and *16S* (top 10 blast results are shown); Table S2: List of 29 sequences used for phylogenetic analysis; Table S3: Original and TrimAl lengths of 13 PCGs, 2 rRNAs, and 13 PCGsAA sequences; Table S4: Best partitioning schemes and models based on different datasets for maximum likelihood and Bayesian inference analysis; Table S5: Codon numbers and relative synonymous codon usage (RSCU) of 13 PCGs in three mitogenomes.
